# Physical stimulation by REAC and BMP4/WNT-1 inhibitor synergistically enhance cardiogenic commitment in iPSCs

**DOI:** 10.1371/journal.pone.0211188

**Published:** 2019-01-23

**Authors:** Valentina Basoli, Sara Santaniello, Salvatore Rinaldi, Vania Fontani, Gianfranco Pigliaru, Matthias Wieser, Agata Strajeriu, Alessandro Castagna, Heinz Redl, Carlo Ventura, Regina Grillari, Margherita Maioli

**Affiliations:** 1 Department of Biomedical Sciences, University of Sassari, Sassari, Italy; 2 Research Department, Rinaldi Fontani Foundation, Florence, Italy; 3 Department of Biotechnology, University of Natural Resources and Life Sciences, Vienna, Austria; 4 National Laboratory of Molecular Biology and Stem Cell Engineering - National Institute of Biostructures and Biosystems-Eldor Lab, at Innovation Accelerators, CNR, Bologna, Italy; 5 Department of Regenerative Medicine, Rinaldi Fontani Institute, Florence, Italy; 6 IRF Shanghai Medical Sciences, Shanghai, China; 7 Evercyte GmbH, Vienna, Austria; 8 Ludwig Boltzmann Institute for Experimental and Clinical Traumatology, Vienna, Austria; 9 Austrian Cluster for Tissue Regeneration, Vienna, Austria; 10 Center for developmental biology and reprogramming - CEDEBIOR, Department of Biomedical Sciences, University of Sassari and National Institute of Biostructures and Biosystems, Sassari, Italy; Macau University of Science and Technology, MACAO

## Abstract

It is currently known that pluripotent stem cells can be committed *in vitro* to the cardiac lineage by the modulation of specific signaling pathways, but it is also well known that, despite the significant increase in cardiomyocyte yield provided by the currently available conditioned media, the resulting cardiogenic commitment remains a highly variable process. Previous studies provided evidence that radio electric fields asymmetrically conveyed through the Radio Electric Asymmetric Conveyer (REAC) technology are able to commit R1 embryonic stem cells and human adipose derived stem cells toward a cardiac phenotype. The present study aimed at investigating whether the effect of physical stimulation by REAC in combination with specific chemical inductors enhance the cardiogenic potential in human induced pluripotent stem cells (iPSCs). The appearance of a cardiac-like phenotype in iPSCs cultured in the presence of a cardiogenic medium, based upon BMP4 and a WNT-inhibitor, was consistently increased by REAC treatment used only during the early fate differentiation for the first 72 hours. REAC-exposed iPSCs exhibited an upregulation in the expression of specific cardiogenic transcripts and morphologically in the number of beating clusters, as compared to cells cultured in the cardiogenic medium alone. Our results indicate that physical modulation of cellular dynamics provided by the REAC offers an affordable strategy to mimic iPSC cardiac-like fates in the presence of a cardiogenic milieu.

## Introduction

Stem cell-based therapy currently represents a promising approach for regenerative medicine, although the translation of the in vitro studies into clinical applications is in part complicated by the difficulty in obtaining a high yield of differentiation within specific and homogenous stem cell-derived lineages that can provide their safety. There is increasing evidence that defined stem cell commitment can be promoted not only by chemical agents[1, 2] but can result from exposure to physical energies. In this regard, we have shown that exposure to Radio Electric Asymmetric Conveyer (REAC) technology with specific treatment protocol, can promote multi-lineage commitment, like cardiogenesis, neurogenesis and skeletal myogenesis, in mouse embryonic stem (ES) cells[3] and in human adipose-derived mesenchymal stem cells (ADhMSCs)[4, 5], also modulating the expression of stemness-related genes and the commitment to cardiac-, neural- and skeletal muscle-like fates in human fibroblasts[6]. Moreover, previous studies have shown that REAC technology can also influence other processes[7], activating both telomerase-dependent and independent pathways to counteract and even reverse stem cell senescence *in vitro*[8–10]. These responses to REAC exposure involved a major role of intracellular hyaluronic acid patterning and the establishment of intracellular networks acting on the modulation of cell polarity [11].

The discovery of induced pluripotent stem cells (iPSCs)[12] has notably transformed previously defined stem cell-based therapeutic approaches[13] showing new applications for regenerative medicine[14], drug discoveries and developmental studies[15]. Many investigators have focused their efforts on developing strategies to efficiently and reliably direct stem cell differentiation towards the cardiovascular lineage [16], holding hopes for future clinical applications [17]. Pluripotent stem cells can be committed in vitro to the cardiac phenotype by the modulation of specific signaling pathways[18], afforded by naturally occurring molecules[19]. Despite the significant increase in cardiomyocyte yield provided by the currently available conditioned media, the resulting cardiogenic commitment[20] remains a variable and problematic process. The present study aims at investigating whether REAC technology may be used in cooperation with specific cardiogenic inductors to turn iPSC cardiogenesis into a high-throughput and reproducible differentiating outcome. Here, REAC exposure has been applied to enhance cardiogenesis in iPSCs obtained from human exfoliated epithelial cells from urine (hUiPSCs) by the aid of non-integrating technologies [21]. Since spontaneous cardiogenesis is a low-yield, variable process even in embryonic stem cells, the current observation that REAC technology with specific treatment protocols can promote the achievement of a cardiac phenotype in hUiPSCs unravels novelty within the methods actually in use to commit stem cells toward cardiac-like cells and opens new perspectives for future clinical applications.

## Materials and methods

### Ethics approval

The hUiPSCs line was developed from urine-derived cells belonging to the same sample used in the study conducted by Zhou et al. in China. The ethics committee of the Guangzhou Institutes of Biomedicine and Health, Guangzhou, China and the University of Natural Resources and Life Sciences, Vienna, Austria approved the previous study. The IRB of the Centre for developmental biology and reprogramming (CEDEBIOR) of University of Sassari, Italy approved this stage of the study.

### Description of Radio Electric Asymmetric Conveyer (REAC) technology

Radio Electric Asymmetric Conveyer Technology for therapeutic use (REAC) is a technology for bio and modulation. The REAC technology generates a radio electric emission of very low intensity. The radio electric emission interacts with all the structures that contain electrical charges, such as the human body, and induces currents in them. These currents vary according to the molecular characteristics of the tissues. The peculiarity of REAC technology is not the radio electric emission itself, but the particular physic link between the device and the patient’s body. The “Asymmetric Conveyer Probe” (ACP) represents this link. This is the innovation of REAC technology. This scheme has been developed for a specific purpose: to create an asymmetric circuit for better interact with the asymmetric mechanism underlying the cell polarity[22], in order to optimize its functions. In fact, REAC technology is able to modulate the current flows existing both at cellular and body level, when these are altered. Another peculiarity of REAC Technology is the low power level used in radio frequency emission. This is necessary to induce current flows of intensity comparable with those of cell polarity. Higher power levels would disturb the adjustment mechanisms of cell polarity. The REAC technology is independent of the radio frequency emission used. REAC devices use only two frequencies (2.4 and 5.8 GHz). These two frequencies were chosen for two reasons. First of all, these are the most widely used and permitted at the international level. Secondly, based on our clinical and scientific experience, the 2.4 GHz frequency was chosen to better interact with tissues and cell cultures [3–7, 10, 23–27], while the 5.8 GHz frequency was chosen to better interact with the nervous system.

The electromagnetic quantities have been measured immerging the REAC ACP into the culture medium, inside a CO_2_ incubator, using the spectrum analyzer Tektronix model 2754p and orienting the receiving antenna for maximum signal. The distance between the device and the culture medium was approximately 35 cm. Radiated power was about 2 mW, electric field E = 0.4 V/m, magnetic field 1 mA/m, specific absorption rate (SAR) 0.128 μW/g; determinate σ = 1 A/V.m and ρ = 1000K g/m3 the density of radio electric current flowing in the culture medium during a single REAC pulse was J = 30 μA/cm2. The REAC treatment protocol used in this study was tissue optimization (TO) regenerative treatments (RGN). The REAC model device used in this study was B.E.N.E (ASMED, Florence, Italy).

### hUiPSC culture

The hUiPSCs line was developed from urine-derived cells as previously described [28]. Cells were routinely grown on Matrigel Matrix (Invitrogen) in mTeSR_1 medium (STEMCELL Technologies) at 37°C degree in an incubator with 5% CO_2_ and 3% O_2_. Cells were subcultured using STEMPRO ACCUTASE (Life Technologies) and ROCK-Inhibitor Y-27632 (Enzo Life Sciences).

### Cardiomyocyte differentiation

Undifferentiated hUiPSCs were digested into smaller clusters using ACCUTASE and seeded onto Matrigel-coated plates at 3 · 10^5^ cells/10 cm^2^ in mTeSR1 medium until they reached 80%–90% confluence. Thereafter, the cells were digested into single-cell suspensions and seeded into ultralow-attachment six-well plates (Corning) and cardiac differentiation was induced as described elsewhere[29]. Briefly, cells were kept for 24 hours in mTeSR1 medium with Matrigel (40 mg/mL), BMP4 (1 ng/mL; Invitrogen) and Rho kinase inhibitor (ROCK) (10 μM; R&D) under a hypoxic condition with 3% O_2_. Then, the first group of cells was washed and replaced in cardiogenic medium (Medium C -REAC), containing: StemPro34 SFM (Invitrogen) with ascorbic acid (AA, 50 mg/mL; Sigma), 2mM Gluta-MAX-1 (Invitrogen), BMP4 (10 ng/mL), and human recombinant activin-A (10 ng/mL; Invitrogen). The second group of cells (Medium C+REAC) was replaced in the presence of the cardiogenic medium and additionally exposed to REAC for 72 hours. On day 4 both groups of cells were incubated with IWR-1, a Wnt inhibitor (5 mM; Enzo Life Sciences). On day 8, all cells were transferred to a normoxic environment and maintained in cardiogenic medium until the end of experiment (day 14). Cardiac mesodermal cells organized in functional contracting clusters were detected as early as day 8.

### RNA extraction, cDNA synthesis, and gene expression analysis by real-time PCR

Total RNA was isolated using Trizol reagent according to the manufacturer’s instructions (Sigma). Total RNA was dissolved in RNAase-free water and quantitated using a Nanodrop.

A twenty-μl reaction volume containing 1 μg total RNA was reverse transcribed using Hi Capacity cDNA reverse transcription kit (Applied Biosystem) with oligo (dT) primers. qPCR reaction was performed using Rotorgene machine (Qiagen) with EvaGreen mix (Biolab).

After an initial denaturation step at 95°C for 15 min, temperature cycling was initiated. Each cycle consisted of 95°C for 15 sec., 53–59°C for 30 s, and 72 °C for 15 sec., the fluorescence being read at the end of this step. All primers used in this work were from Invitrogen and are listed in Table 1.

**Table 1:** Primers used in this work.

PRIMER NAME	REVERSE	FORWARD
*ACTN2*	ACCAGTTTCACCCCTTTGCT	CGCCATGAACCAGATAGAGCC
*GATA4*	CTCAGATCCTTAGGTGCTAGA	TCCTCTTGCCTGGTAATGACTCC
*hMEF2C*	AGTGAGCTGACAGGGTTGCT	GCCCTGAGTCTGAGGACAAG
*hMHCα*	TTTGATGCGCCCGAACTCTT	GAGGAAATGAGGGACGAGAGG
*nkx2*.*5*	TAATCGCCGCCACAAACTCTCC	TATAACGCCTACCCCCGCCTAT
*TBX5*	GTGGGGAGCCATGGTTGGCC	CAGAGTCGGCACAGCGGCAA
*cTnT*	CGTCTCTCGATCCTGTCTTTG	CATGGAGAAGGACCTGAATGA
*GAPDH*	GACAAGCTTCCCGTTCTCAG	GAGTCAACGGATTTGGTCGT

PCR products were confirmed by melting curve analysis and electrophoresis. All measurements were done as technical quadruplicate of biological triplicates. Biological replicates were obtained from independent differentiation setup. Relative expression was determined using “2^ΔΔCt method” with glyceraldehyde 3-phospate dehydrogenase (GAPDH) as normalizing expression levels.

### Immunostaining

hUiPSCs (Embryo bodies clusters), cultured in the cardiogenic medium (Medium C) with or without REAC exposure for the first 72 hours, were digested at day 14 by collagenase 1 (Invitrogen) for 1 hour at 37°C, and the resulting suspension was cultured at low density to visualize individual cells. The cultures were fixed with absolute methanol for 30 min and 1 hour at -80°C. Cells were then exposed to antibodies against α-sarcomeric actinin (Mouse, Sigma, Clone EA-53), Cardiac troponin T (Mouse, Thermo scientific, 1F11) GATA4 (Mouse, Santa Cruz, sc-25310), MEF2C (Rabbit, Thermo scientific, PA5-28247) or to TBX5 (Rabbit, Thermo scientific, PA5-29845) for 1 hour at 37°C, followed by staining with the respective secondary antibodies anti mouse Alexa fluor 594 or anti rabbit Alexa fluor 498 at 37°C for 1 h. All microscopy was performed with a Leica confocal microscope (LEICA TCSSP5). DNA was visualized with DAPI (1 μg/ml).

### Counting of beating cells

Cells seeded in ultralow-attachment six-well plates (Corning) aggregated in EBs clusters were examined under an inverted live microscope (Leika) at 37°C at different time points. In each well (9.5 cm^2^) total EBs were counted at a field with an area of 1 mm^2^. Ten to fifteen fields in each well were randomly chosen and counted. The percentage of contractile EBs was determined as the number of EBs that showed spontaneous contraction divided by the total number of EBs plated.

### Immunoblotting

Cells were cultured in cardiogenic medium (MEDIUM C) in the presence or absence of REAC for 72 hours. Total cell lysates from hUiPSCs committed with cardiogenic medium with or without REAC were electrophoresed on 10% Novex Tris-glycine polyacrylamide gels (Invitrogen) in MOPS sodium dodecyl sulfate running buffer using an XCell SureLock Mini-Cell (Invitrogen) according to the instructions provided by the manufacturer. After protein transfer to polyvinylidenedifluoride membranes (Invitrogen), membranes were saturated and washed, immunoreaction was carried out for one hour at room temperature in the presence of the primary antibody, antisera against Cardiac troponin T (Mouse, Thermo scientific, 1F11), GAPDH (Mouse, Thermo scientific) diluted to 1:1000. After additional washing, membranes were incubated with antirabbit α-Sarcomeric (Rabbit, Santa Cruz). Targeted protein expression was assessed using a chemiluminescence detection system (ECL Western blotting detection reagents were from Amersham Biosciences Corporation, Piscataway, NJ, USA). Densitometric analysis was performed on the images using ImageJ software Fiji extension. We analyzed the densitometry of each band for cTnT and α-Sarc using GAPDH as reference protein.

## Data analysis

### Statistical analysis

Statistical analysis was performed using GraphPad Prism 7.03 software; this software gives a corrected p-value for multiple comparisons by comparing means regardless different time point and the treatment with or without REAC for the different biological replicate. For this study, the non-parametric ANOVA two-way and in conjunction Sidak’s multiple comparison test were applied. The tests and results with p < 0.05 (95% confidence interval) were considered statistically significant considering values of interaction, Row factor and column factor below 0.05 as limit of significance, with The ANOVA two-way was used to evaluate the distributions and homogeneity variance in the groups among undifferentiated hUiPSCs and cells committed to cardiogenesis in the absence (Medium C alone) or presence of REAC.

## Results

### Evaluation of UIPSCs features in culture

**Fig 1:**
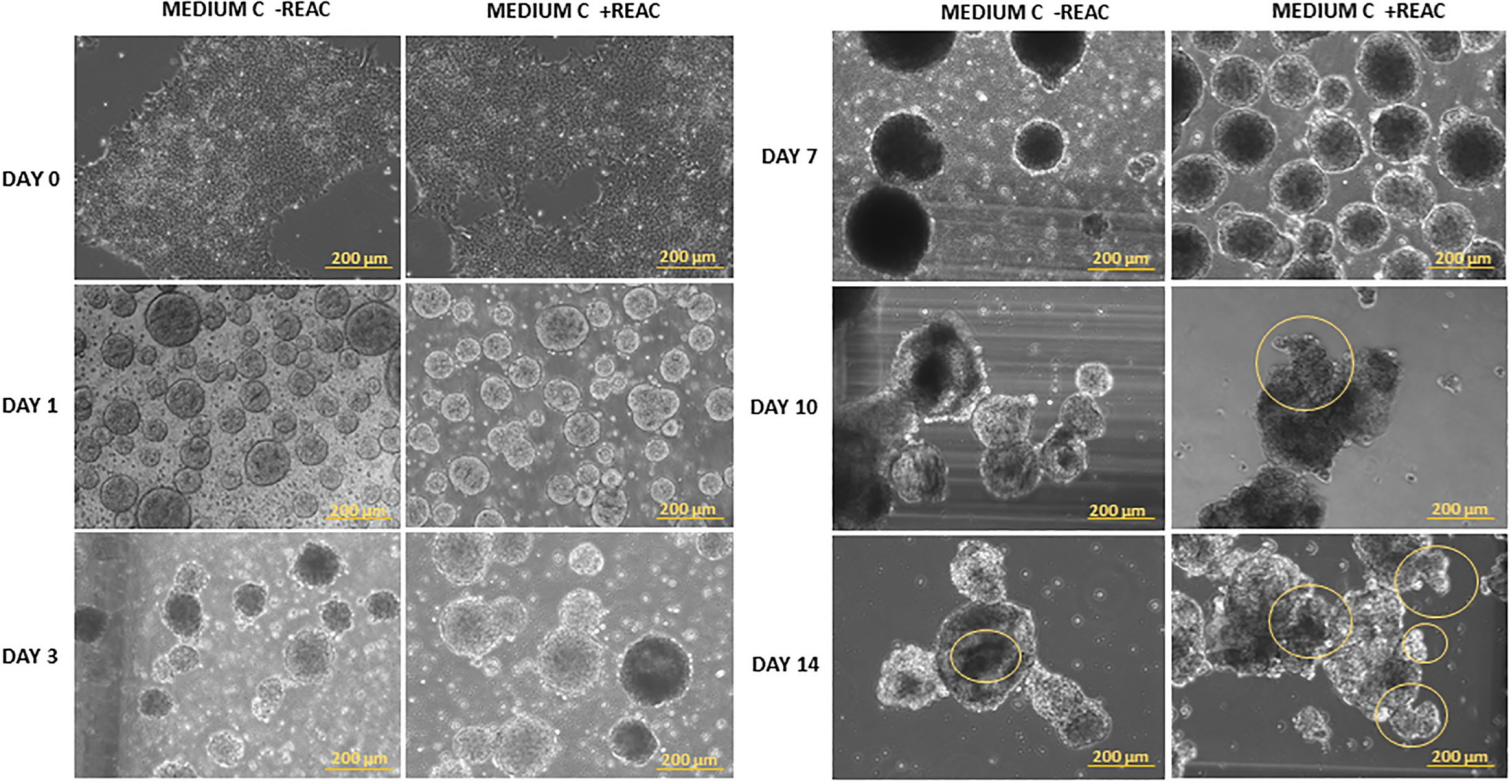
Representative morphologycal evaluation of hUiPSCs over the time in supplemented defined medium and REAC. hUiPSC at day 0 and during the differentiation, culturing in 3D condition in cardiogenic conditioned medium in the absence (Medium C-REAC) or presence of REAC (Medium C+REAC). The yellow circles in the pictures represent the beating cluster zones. The yellow scale bars are 200 μm.

iPS cells culture is apparently simple and not so different from the culture of other cell types. Yet, it has the peculiarity of the instability. In fact, although cultured with rigorous and reproducible methods, these cells very often tend to autonomously differentiate. That means that if used for differentiation in experiments, iPS cells could result in consistently different outcomes of differentiation. On this basis, for each experiment the morphology and probable self-differentiation (Fig 1) was evaluated throughout the 14 days of culturing.

**Fig 2:**
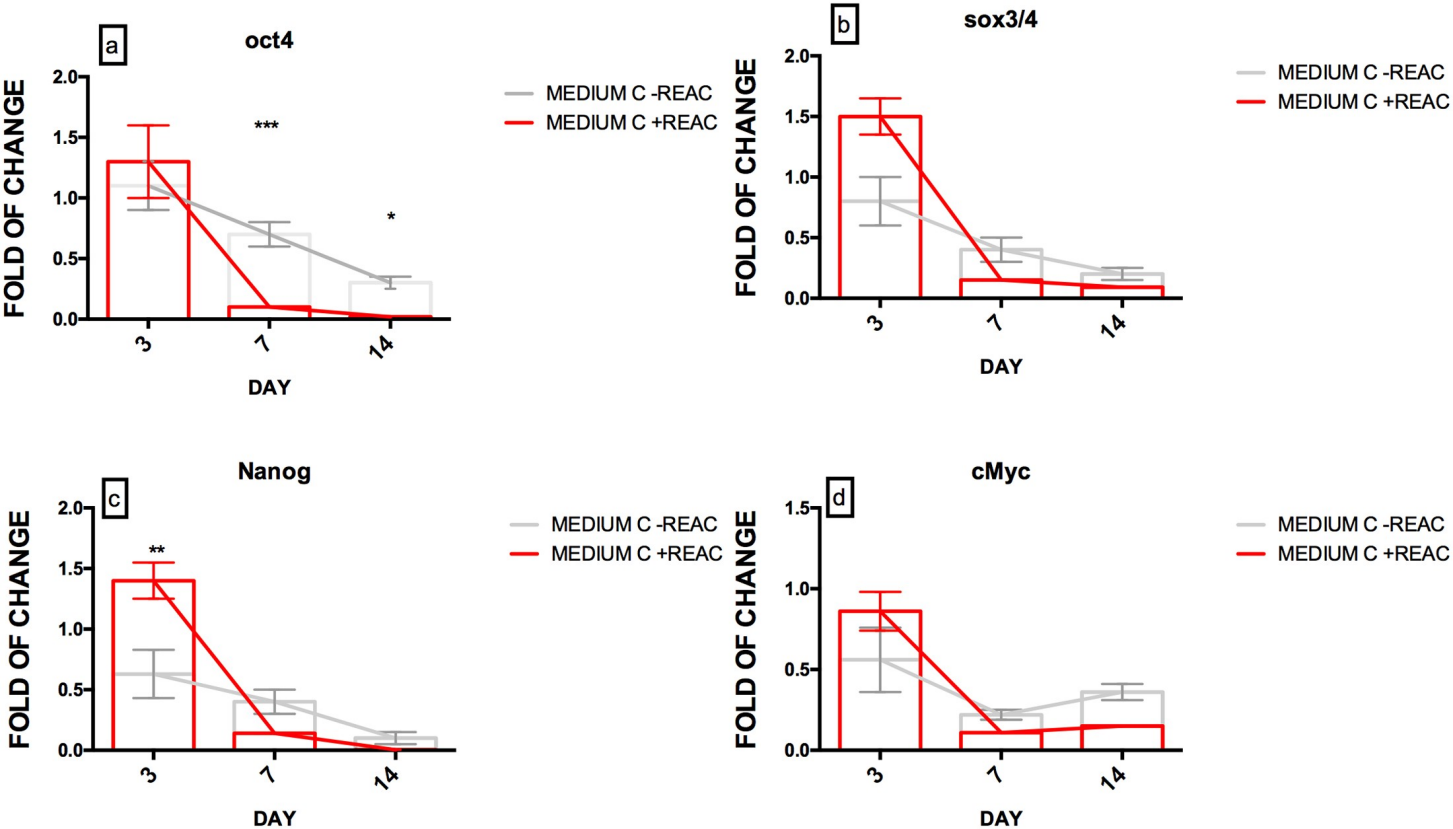
Effect of REAC treatment on the expression of pluripotency genes in hUiPSCs supplemented with defined medium. Cells cultured in cardiogenic conditioned medium (Medium C) were exposed for 72 h, in the absence (grey bars) or presence (red bars) of REAC and then cultured in the presence of medium C alone until day 14th. The amount of OCT4(A), SOX3/4 (B), NANOG (C) and cMyc (D) mRNA from untreated (grey bars) or REAC-treated (red bars) cells was normalized to glyceraldehyde 3-phosphate dehydrogenase (GAPDH) and was plotted as fold of change relative to the mRNA expression in hUiPSCs at day 0. Data shown mean±S.D; n = 4 (P<0.05 **), (P<0.001* **).

We have also analyzed the expression of the pluripotency genes at day 0 before the commitment and during the 14 days of differentiation in culture, to detect changing in the molecular differentiation program through the downregulation of OCT, SOX3 / 4, NANOG and cMyc (Fig 2).

### REAC-RGN treatment protocol enhances the expression of the cardiogenic and cardiac specific genes

**Fig 3:**
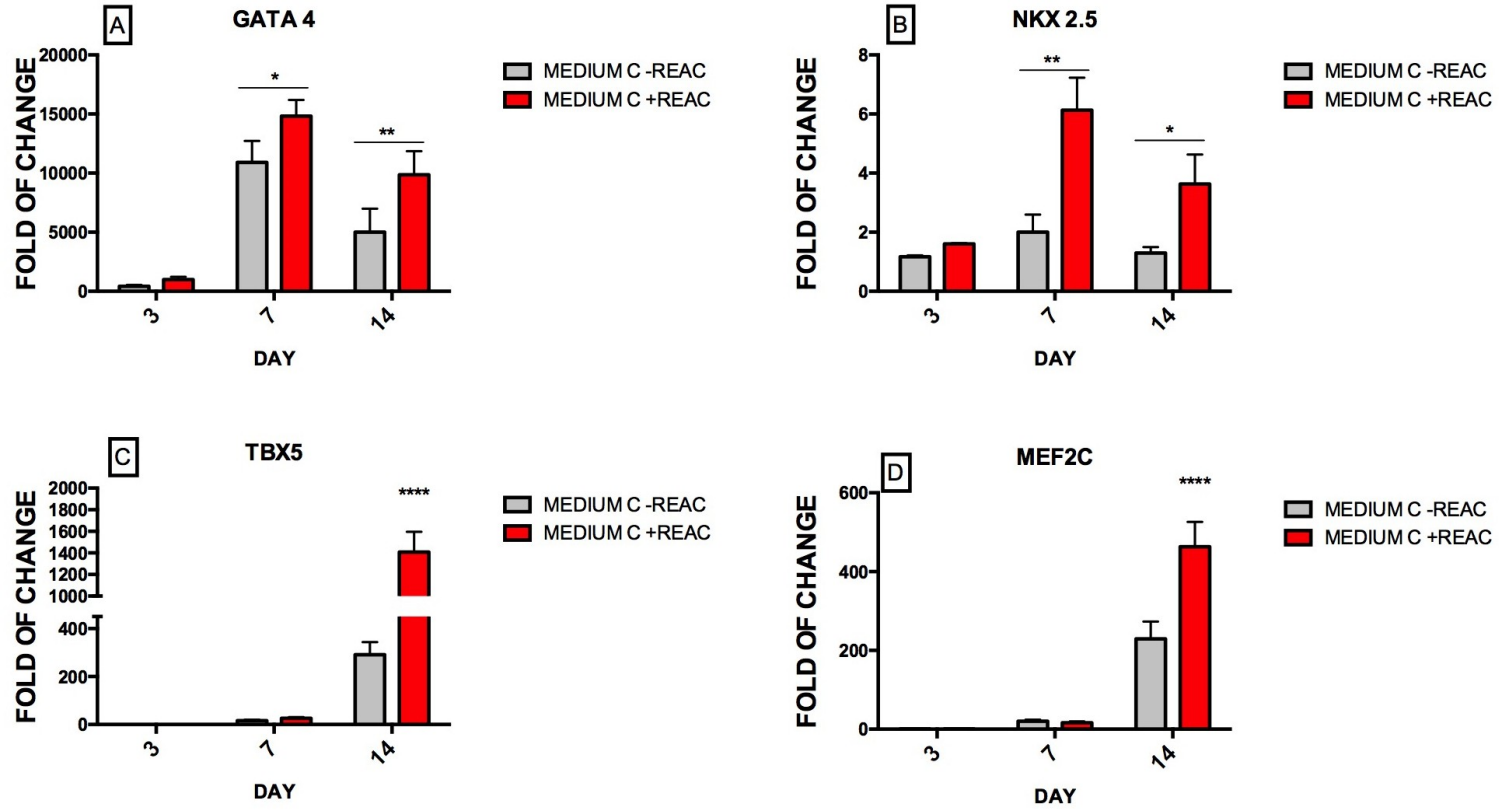
Effect of REAC treatment on the expression of cardiogenic transcripts in hUiPSCs supplemented with defined medium. Cells cultured in cardiogenic conditioned medium (Medium C) were exposed for 72 h, in the absence (grey bars) or presence (red bars) of REAC and then cultured in the presence of medium C alone until day 14th. The amount of GATA4 (A), Nkx-2.5 (B), Tbx5 (C) and Mef2C (D) mRNA from untreated (grey bars) or REAC-treated (red bars) cells was normalized to glyceraldehyde 3-phosphate dehydrogenase (GAPDH) and was plotted as fold of change relative to the mRNA expression in hUiPSCs at day 0. Data shown mean±S.D; n = 4 (P<0.05 **), (P<0.001* **).

REAC-RGN exposure of hUiPSCs in the presence of Medium C remarkably enhanced the transcription of the early cardiogenic markers Nkx2.5 and GATA4, encoding a homeodomain and a zinc finger essential in early cardiogenesis, as compared to expression levels achieved in the presence of the cardiogenic medium alone (Fig 3), that seem actively regulated in the presence of cardiogenic medium containing BMP4 and the WNT-1 inhibitor compared to starting point IPS cells at day 7 but not at day 3.

Gene expression levels of both T-Box protein 5 (TBX5) and MADS box transcription enhancer factor 2 (MEF2), pivotal proteins during cardiogenic commitment, were also dramatically increased in hUiPSCs that had been cultured in Medium C in the presence of the physical modulation by REAC at 14 days.

**Fig 4:**
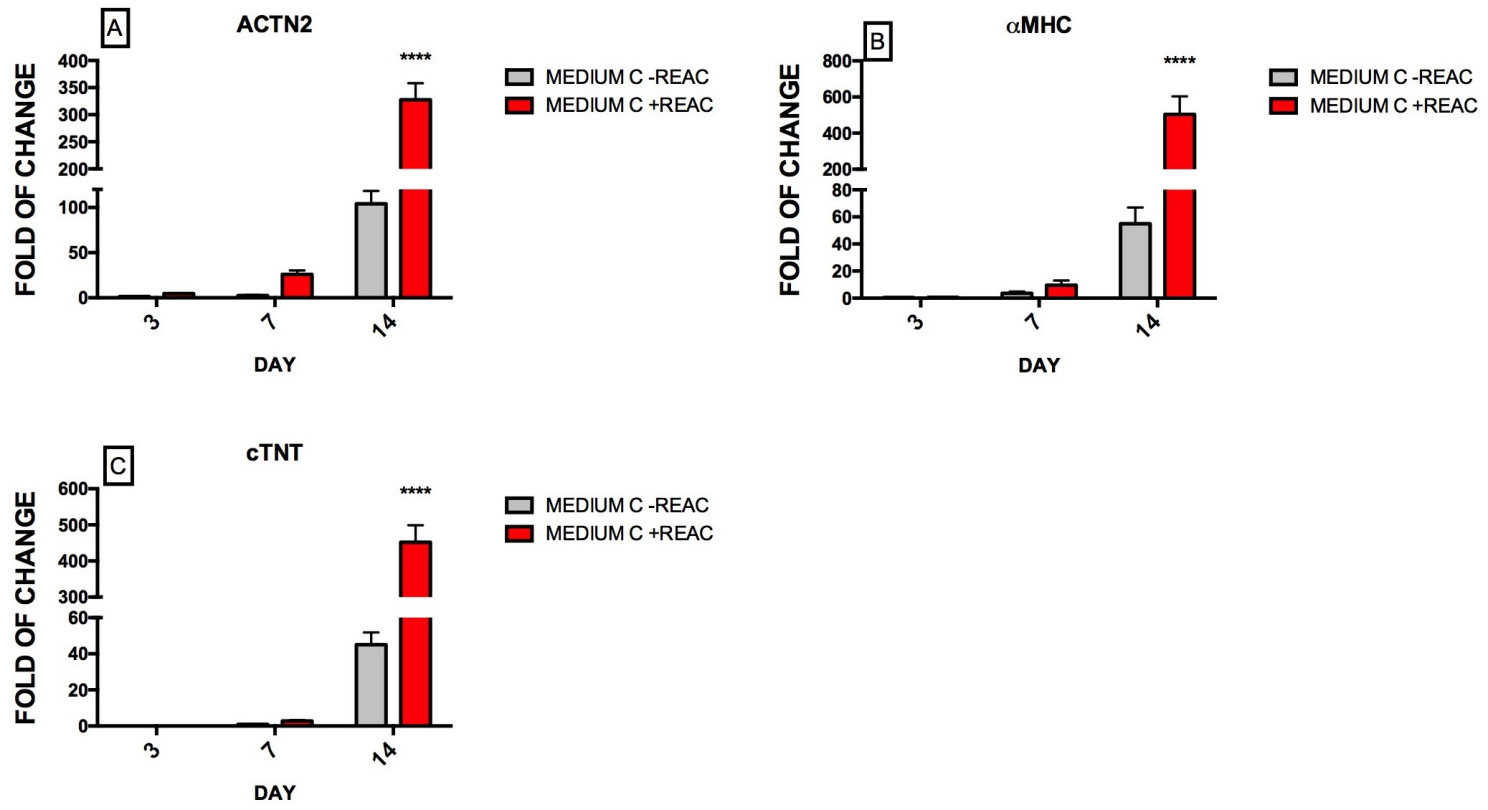
Effect of REAC treatment on the expression of early cardiac-specific marker genes in hUiPSCs supplemented with defined medium. Cells cultured in cardiogenic conditioned medium (Medium C) were exposed for 72 hours, in the absence (grey bars) or presence (red bars) of REAC, and then cultured in the presence of medium C alone until day 14. The amount of ACTN2 (A), αMHC (B), and cTnT (C) mRNA from untreated (grey bars) or REAC-treated (red bars) cells was normalized to glyceraldehyde 3-phosphate dehydrogenase (GAPDH) and was plotted as fold of change relative to the mRNA expression of hUiPSCs at day 0. Data shown mean±S.D; n = 4 (P<0.05 **), (P<0.001* **).

The use of physical energies in combination with the cardioinductive compounds consistently enhanced the transcription of genes underlying a terminal cardiac differentiation, including ACTN2, encoding an actin-binding protein, alpha-myosin heavy chain (MHC) and the Troponin complex (cTnT), encoding two major cardiac sarcomeric proteins. These effects were detected as early as 72 h and peaked after 14 days, underling a strong and mature differentiation on the molecular activation way (Fig 4).

### REAC-RGN treatment protocol drives the expression of cardiac specific marker proteins

**Fig 5:**
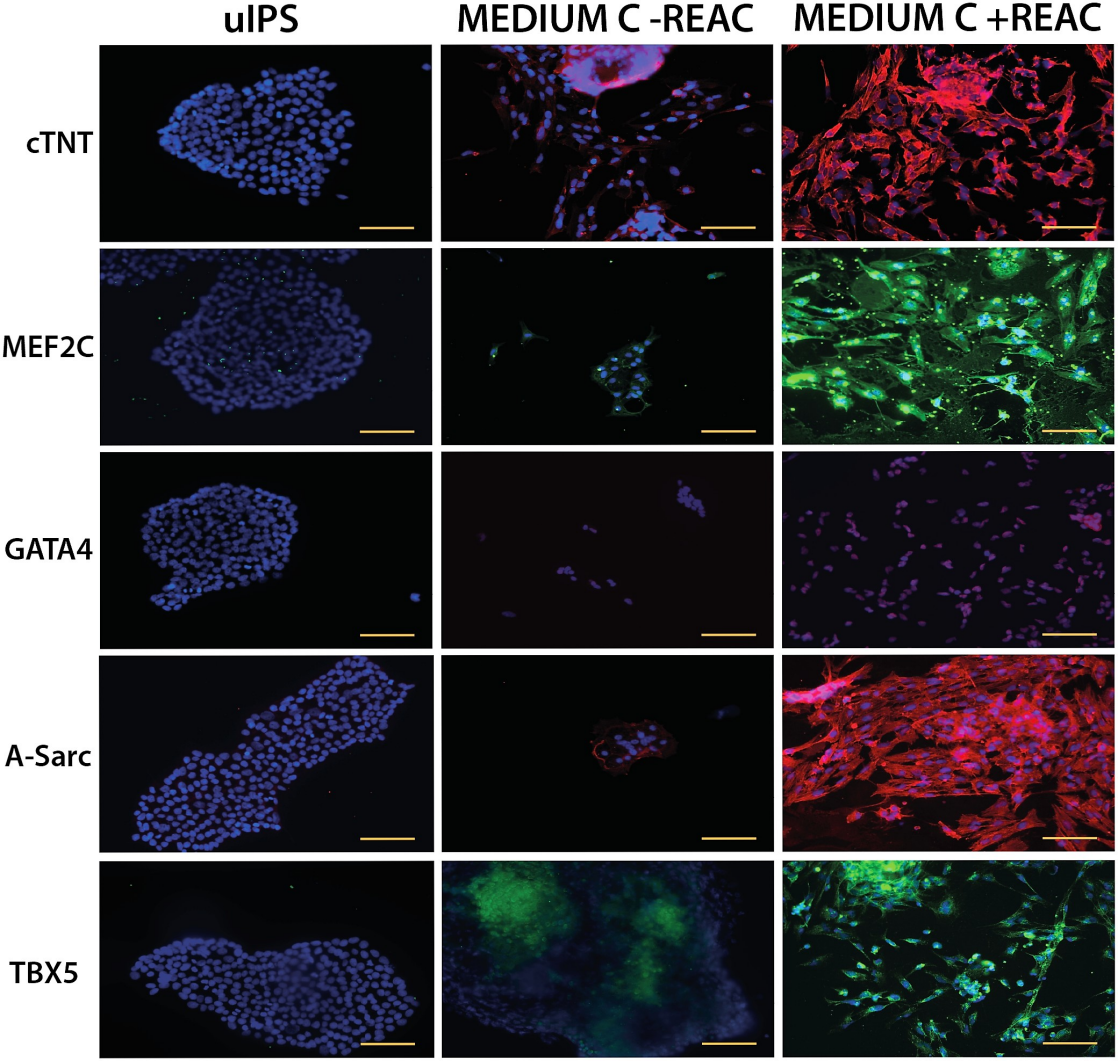
Immunohistochemical analysis of cardiac specific proteins. hUiPSCs exposed for 72 hours to conditioned medium in the absence (Medium C-REAC) or presence of REAC (Medium C+REAC) were disaggregated after 14 days from time 0 (undifferentiated hUiPSCs) seeded in chamber slide and processed for immunostaining using specific antibodies directed against: Troponin complex (cTnT), MADS box transcription enhancer factor 2 (MEF2C), GATA binding protein 4 (GATA4), α-sarcomeric actinin (A-Sarc) and T-Box protein 5 (TBX5). Confocal microscopy analysis was performed with Leica confocal microscope (LEICA TCSSP5). Nuclei were labeled with DAPI (blue). The yellow scale bars are 100 μm.

However, to prove the effectiveness of the synergic action on the UiPSCs by REAC-RGN and BMP4, WNT-1 inhibitor even on the protein level, confocal microscopy analysis was performed in order to validate molecular gene expression observations. Interestingly, REAC-RGN exposure resulted in a consistent increase in the yield of cells positively stained for the cardiac specific marker proteins cTnT, accord with gene expression activation. As well, MEF2C, α-sarcomeric and TBX5, as compared to cells that had been cultured with Medium C alone (Fig 5) confirm again transcriptional observations.

**Fig 6:**
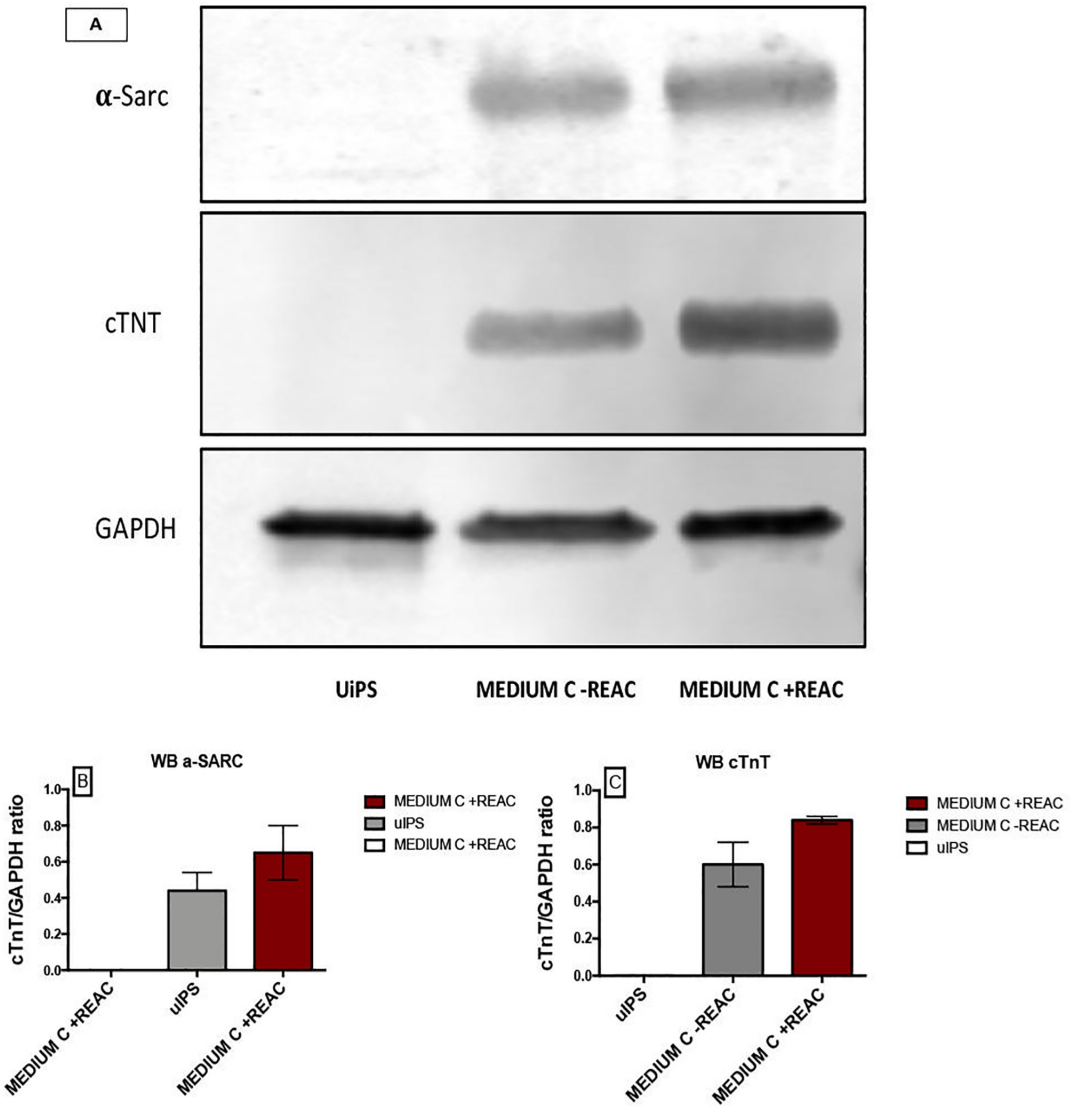
Effect of REAC treatment on the expression of cardiogenic markers in hUiPSCs after 14 days. Total lysates were isolated from hUiPSCs committed by Cardiogenic medium (Medium C) in the absence or presence of REAC treatment for 72 hours. Samples were analyzed by Western blot, using antisera against α-Sarcomeric actinin (α-Sarc), cardiac troponin T (cTNT) and GAPDH (A). The sizes of the bands were determined using prestained marker proteins. The data presented are representative of five separate experiments. Two different membranes were incubated with anti-mouse (cTNT, GAPDH) or anti-rabbit (α-Sarc), densitometry analysis for α-Sarc (B) and cTnT (C) were performed with ImageJ.

As expected for terminally differentiated myocardial cells, the expression of GATA4, an early cardiogenic transcription factor, was only faintly detectable in REAC-RGN exposed cells (Fig 5). These results were further inferred by western blot analysis of the specific cardiac protein α-sarcomeric actinin and cTNT (Fig 6).

### REAC-RGN treatment maximizes the yield of spontaneously beating cardiomyocytes

**Fig 7:**
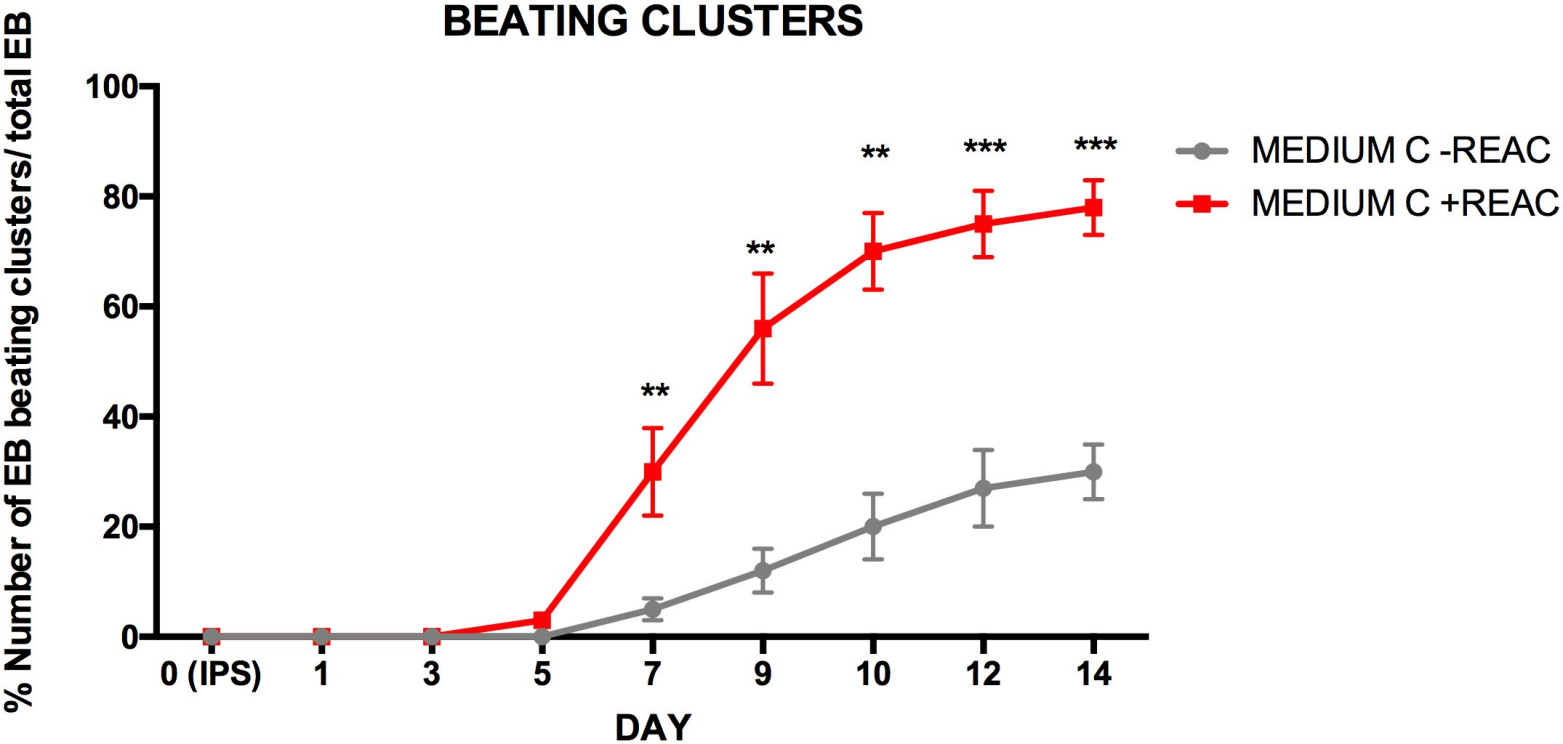
Analysis of the beating clusters. The Figure shows the analysis of the yield of total spontaneously beating clusters obtained from hUiPSCs aggregated as EBs grown in cardiogenic conditioned medium in the absence (continuous grey line) or presence of REAC (continuous red line). Also shown is the percentage of beating clusters, assessed as the number of EBs that showed spontaneous contraction divided by the total number of EBs plated in cardiogenic conditioned medium in the absence (discontinuous grey line) or presence of REAC (discontinuous red line), and then assayed at the indicated time periods for the appearance of a contractile activity (mean ± SE; n = 4).

The achievement of a cardiac phenotype was remarkably confirmed by the observation that REAC-RGN exposure of Medium C-supplemented cells consistently increased the number of beating clusters of hUiPSCs-derived cardiomyocytes. In particular, the exposure to REAC-RGN treatment anticipated the appearance of beating clusters at day 8th, while a consistent number of beating clusters could be observed at day 14th in Medium C- supplemented hUiPSCs (Fig 7).

At day 10th, more than 100 beating clusters/well were consistently observed, with a further progressive increase at later observational times. On the contrary, as shown in Fig 5, a far lesser number of beating clusters was detected from cells that had been cultured in the presence of Medium C alone.

## Discussion

In the field of regenerative medicine iPSCs hold a high promise for future development, owing to its easy and wide-ranging opportunities for harvesting, and for the possibility of being generated even by non-integrating technologies[21], however one of the main issues is to find a way to improve their differentiation in order to create a stable population for further applications.

We previously demonstrated that the use of physical energies by REAC-RGN in R1 mouse embryonic stem cells during the early stage of cardiac commitment was able to modulate the expression of GATA4 and NKX-2.5 genes, two pivotal transcription factors orchestrating cardiogenesis, thus promoting the appearance of a cardiac phenotype. Within this context, other authors also showed that electromagnetic fields, another kind of physical stimulus, committed embryonic stem cells to cardiogenesis by modulating ROS production, and intracellular ion concentrations[30–32] [33].

In the present paper, we aimed to figure out the behavior of hUiPSs exposed to REAC-RGN during the cardiogenic differentiation in the presence of BMP4 and WNT-1 inhibitor. Our results showed that the use of physical stimulation by REAC in combination with cardioinductive medium was able to increase the modulation of transcription factors genes GATA4, NKX-2.5, TBX5, mainly involved in cardiac fate in the early three days of differentiation. This trend was maintained during time, acting on the mature differentiation in beating cardiomyocytes after eight days in culture under the differentiating stimuli. These observations were further inferred by the study of both gene expression and protein levels (immunohistochemistry) of cTnT, MEF2C, α-sarcomeric, cardiac specific proteins expressed only in mature myocytes. Modulation of stem cell fate is an extremely fascinating mechanism finely controlled by an uncountable number of variabilities still not well defined. However, here we demonstrate that the use of REAC conveyed radioelectric fields can interact with this fate, and hypothesize that cardiogenesis can be turned into a high-throughput process by a main mechanism elicited by physical energies in combination with specific compounds. To this end, we cannot exclude the involvement of ROS production and the activation of a WNT non-canonical pathway. Indeed, it has been previously demonstrated that redox-based mechanisms can have a central role in cardiogenic differentiation[34] and in beating cluster formation. Recent studies indicated the potential involvement of the transmembrane enzyme NADPH oxidases (Noxs), in particular Nox4, which catalyzes the reduction of molecular oxygen to superoxide anion, in this process [35, 36]. Within this context, we have previously demonstrated the direct involvement of NADPH oxidase, and ROS signaling during the direct cell reprogramming induced by REAC[6]. The use of physical energies, in combination with specific growth factors like BMP4 and the use of WNT-1 inhibitor apparently increase the cardiogenic commitment. It has been largely described, that non-canonical or β-catenin-independent WNT pathway orchestrates planar cell polarity by cytoskeleton reorganization[37]. We have previously shown that the molecular effect elicited by REAC on adipose derived stem cells involved the synthesis of hyaluronic acid, which controls cell growth and polarity, and thus stem cell capability to acquire a specific phenotype[11].

Here, by the chemical block of WNT-1 we induced cardiogenesis probably promoting the non-canonical activation pathway. From our results and previous findings we can postulate that the electromagnetic and radio electric field energies emitted by REAC during the first three days of cardiogenic commitment may recapitulate the physiological behavior of ES cells during cardiogenesis through the WNT-non canonical pathway modulation[38].

The discovery of iPSCs has surely opened new ways and perspectives in the field of regenerative medicine, in particular in personalized medicine. From the pharmaceutical point of view, the discovery of iPSCs has allowed the creation of specific cellular models addressed to the understanding of specific diseases. However, finding a protocol and a method that can allow a high and reproducible yield is still an open issue. Cell differentiation is one of the crucial points still under study. The focus is trying to create a more physiological environment for cells, both at the molecular, and possibly physical level. These stimuli should have the capability of mimicking the physiological environment by increasing the activation of specific pathways involved in phenotypic maturation of cells and tissues.

The hostile inflammatory environment typical of post-infarction necrotic tissue together with the lack of oxygen in the ischemic area may limit the survival and proliferation of endogenous progenitor cells, possibly promoting scar formation and even switching endogenous stem cells to a fate of fibrosis[39]. Hence, a novel strategy could be boosting endogenous mechanisms of regeneration by the identification of a specific protocol, leading to improved cellular resistance to oxidative stress and to increase proliferation and commitment of progenitor populations to cardiomyocyte and other cardiopoietic lineages, to repair the injured tissue[40].

We have previously shown that REAC is able to modulate stem cell pluripotency acting as a hormetic factor, able to strengthen stem cell behavior in inflammatory conditions. Combining molecules that can induce cardiogenesis, and at the same time creating a favorable milieu in which the same cells can acquire a specific phenotype despite the hostile environment will represent in the next future a great breakthrough to clinical studies.

Our finding that REAC technology can unfold a gene and protein expression program promoting an increase in the yield of spontaneous beating cardiac-like cells prompts the chance to circumvent the extremely low-yield of spontaneous cardiogenesis, so far, a considerable drawback in stem cell based regenerative medicine, drug discovery and developmental biology studies.

## Supporting information

S1 File Approval of the study by the IRB of the Centre for developmental biology and reprogramming (CEDEBIOR) of University of Sassari. (PDF)

## Abbreviations

ACP: Asymmetric Conveyer Probe
ADhMSCs: human adipose-derived mesenchymal stem cells
HUiPSCs: human urine induced pluripotential stem cells
iPSCs: induced pluripotent stem cells
REAC: Radio Electric Asymmetric Conveyer technology
