# Supplementary material for: Physical stimulation by REAC and BMP4/WNT-1 inhibitor synergistically enhance cardiogenic commitment in iPSCs
Source: PLoS One. 2019 Jan 23;14(1):e0211188. doi: 10.1371/journal.pone.0211188 (PMC6343882; doi:10.1371/journal.pone.0211188)
Supplement: S1 File — (PDF) [file pone.0211188.s001.pdf]

uniss
DIPARTIMENTO DI SCIENZE BIOMEDICHE
viale San Pietro, n. 43/B, 07100 Sassari (Italy)
t +39 079 228514/8583
f +39 079 228620
dip.scienze.biomediche@pec.uniss.it
www.uniss.it

CENTRO PER LA BIOLOGIA DELLO SVILUPPO E DELLA RIPROGRAMMAZIONE CELLULARE (CENTRE FOR DEVELOPMENTAL BIOLOGY AND REPROGRAMMING-CEDEBIOR)

Sassari, 19 Gennaio 2018
VERBALE N.012/2018

Durante la riunione svoltasi il giorno 19 Gennaio 2018, l'IRB del CEDEBIOR esprime parere positivo allo studio scientifico presentato dalla Dr.ssa Valentina Basoli, finalizzato ad investigare l'efficacia di una stimolazione fisica, somministrata nello specifico con la tecnologia REAC, nell'incrementare il potenziale cardiogenico in cellule staminali umane pluripotenti indotte (iPSCs), coltivate in medium cardiogenico.
Per lo studio in oggetto, è previsto l'utilizzo di materiale biologico derivante da campioni prelevati per studi analoghi condotti con l'approvazione del comitato etico della struttura di riferimento (Guangzhou Institutes of Biomedicine and Health, Guangzhou, China e University of Natural Resources and Life Sciences, Vienna, Austria). Non è richiesto quindi il consenso informato dei donatori.

During the meeting held on 19th January 2018, the IRB of CEDEBIOR expresses a positive opinion to the scientific study presented by Dr. Valentina Basoli, aimed to investigate the efficacy of a physical stimulation, specifically administered by REAC technology, in enhancing the cardiogenic potential in human induced pluripotent stem cells (iPSCs), cultured in the presence of a cardiogenic medium.
For the study in question, biological material will be used deriving from samples taken for similar studies conducted with the approval of the ethics committee of the reference structures ((Guangzhou Institutes of Biomedicine and Health, Guangzhou, China and the University of Natural Resources and Life Sciences, Vienna, Austria). Therefore, the informed consent of donors is not required.

Il Coordinatore
Prof. Pier Andrea Serra
